# Prevalence and Comparison of the Profile of Patients Who Did Not Attend (DNA) Their Appointments Through Face-Face, Pre-Pandemic vs. Telephonic Consultations, Pandemic at the Complex Care Service- South, Wolverhampton - a Service Evaluation

**DOI:** 10.1192/bjo.2022.399

**Published:** 2022-06-20

**Authors:** Pooja Kuppili, Jeyagajani Jeyanathan, Nwamaka Odogwu, Amina Rashid, Susmit Roy

**Affiliations:** 1 Berkshire Healthcare NHS Foundation Trust, Reading, United Kingdom; 2 The Royal Wolverhampton NHS Trust, Wolverhampton, United Kingdom; 3 Black Country Healthcare NHS Foundation Trust, Wolverhampton, United Kingdom

## Abstract

**Aims:**

Patients who do not attend the appointments (DNA) pose a significant financial burden on the health care system. During the COVID-19 pandemic, there has been a shift from face-face to telephonic consultations. Our hypothesis was that pandemic can affect the prevalence of profile of clients who DNA. With this background, the aim of the current service evaluation was to assess the prevalence and profile of patients who did not attend their appointments through face-face consultation and telephonic consultations at the Complex care service south [CCS-South], Wolverhampton over a period of one year.

**Methods:**

Retrospective evaluation of records of DNA appointments at CCS- South, Wolverhampton of Face-face appointments during March 17th 2019- March 16th 2020 [Pre-pandemic group] and telephonic consultations during March 17th 2020 – March 16th 2021 [pandemic group] was done. Prevalence of DNA was calculated as the number of DNA in CCS South/ number of DNA in total CCS × 100. Student t test, Mann U Whitney test, Chi square test were used to analyse the data.

**Results:**

The number of DNA in the pandemic group (625) was significantly higher than the pre-pandemic group (376) [χ2 = 86.31, p <0.00001]. Males had significantly higher DNA in pre-pandemic group (59.52%) whereas females had significantly higher DNA in the pandemic group (66.76%) [χ2 = 72.97, p <0.00001]. The mean (SD) age of clients in the pandemic group was 41.17 (12.39) years was significantly lower than the mean (SD) age of clients in the pre-pandemic group, 42.87 (13.72) years [t = 1.97; p = 0.049].

There was an increase in the Caucasian British (49.73% vs 38.40%) and Asian (29.52% vs 26.41%) in the pandemic group compared to pre-pandemic group. There was a decrease in the African-Caribbean group (10.11% vs 16.11%) and the mixed/other/unstated group (10.64% vs 19.20% vs) in the pandemic group compared to pre-pandemic group. It was the first DNA for twenty-four service users in the pre-pandemic group and none in the pandemic group. Those in the pandemic group (6.39 (6.79)) had significantly higher mean (SD) number of previous DNA than the pre-pandemic group (5.41 (7.50) [U = 98145; W = 293770; Z= -4.40; p = 0.000). There was no significant difference between the time of the appointment in both groups.

**Conclusion:**

There was an increased number of DNA during the pandemic period and the profile of those who DNA during the pandemic was of that a female with a mean age of about 41 years with previous DNAs.

